# Polarization of Human Monocyte-Derived Cells With Vitamin D Promotes Control of *Mycobacterium tuberculosis* Infection

**DOI:** 10.3389/fimmu.2019.03157

**Published:** 2020-01-22

**Authors:** Jagadeeswara Rao Muvva, Venkata Ramanarao Parasa, Maria Lerm, Mattias Svensson, Susanna Brighenti

**Affiliations:** ^1^Department of Medicine, Center for Infectious Medicine (CIM), ANA Futura, Karolinska Institutet, Stockholm, Sweden; ^2^Department of Clinical and Experimental Medicine, Linköping University, Linköping, Sweden

**Keywords:** *Mycobacterium tuberculosis*, tuberculosis, macrophages, vitamin D3, immune polarization

## Abstract

**Background:** Understanding macrophage behavior is key to decipher *Mycobacterium tuberculosis* (Mtb) pathogenesis. We studied the phenotype and ability of human monocyte-derived cells polarized with active vitamin D [1,25(OH)_2_D_3_] to control intracellular Mtb infection compared with polarization of conventional subsets, classical M1 or alternative M2.

**Methods:** Human blood-derived monocytes were treated with active vitamin D or different cytokines to obtain 1,25(OH)_2_D_3_-polarized as well as M1- and M2-like cells or fully polarized M1 and M2 subsets. We used an *in vitro* macrophage Mtb infection model to assess both phenotype and functional markers i.e., inhibitory and scavenger receptors, costimulatory molecules, cytokines, chemokines, and effector molecules using flow cytometry and quantitative mRNA analysis. Intracellular uptake of bacilli and Mtb growth was monitored using flow cytometry and colony forming units.

**Results:** Uninfected M1 subsets typically expressed higher levels of CCR7, TLR2, and CD86, while M2 subsets expressed higher CD163, CD200R, and CD206. Most of the investigated markers were up-regulated in all subsets after Mtb infection, generating a mixed M1/M2 phenotype, while the expression of CD206, HLADR, and CD80 was specifically up-regulated (*P* < 0.05) on 1,25(OH)_2_D_3_-polarized macrophages. Consistent with the pro-inflammatory features of M1 cells, Mtb uptake and intracellular Mtb growth was significantly (*P* < 0.01–0.001 and *P* < 0.05–0.01) lower in the M1 (19.3%) compared with the M2 (82.7%) subsets 4 h post-infection. However, infectivity rapidly and gradually increased in M1 cells at 24–72 h. 1,25(OH)_2_D_3_-polarized monocyte-derived cells was the most potent subset to inhibit Mtb growth at both 4 and 72 h (*P* < 0.05–0.01) post-Mtb infection. This ability was associated with high mRNA levels of pro-inflammatory cytokines and the antimicrobial peptide LL-37 but also anti-inflammatory IL-10, while expression of the immunosuppressive enzyme IDO (indoleamine 2,3-dioxygenase) remained low in Mtb-infected 1,25(OH)_2_D_3_-polarized cells compared with the other subsets.

**Conclusions:** Mtb infection promoted a mixed M1/M2 macrophage activation, and 1,25(OH)_2_D_3_-polarized monocyte-derived cells expressing LL-37 but not IDO, were most effective to control intracellular Mtb growth. Macrophage polarization in the presence of vitamin D may provide the capacity to mount an antimicrobial response against Mtb and simultaneously prevent expression of inhibitory molecules that could accelerate local immunosuppression in the microenvironment of infected tissue.

## Introduction

As primary reservoirs for intracellular growth and persistence of *Mycobacterium tuberculosis* (Mtb), macrophages play a critical role in the pathogenesis of tuberculosis (TB) disease. Macrophages comprise a heterogeneous population of cells that are involved in the induction of innate as well as adaptive TB immunity and also contribute to tissue remodeling (1). Polarization of macrophage-like cells from monocytes into phenotypically and functionally different cells occur in response to microenvironmental signals (2, 3) that may affect the ability of the cells to control Mtb infection. Although macrophage activation is complex involving a non-linear range of functional states without clear boundaries (4), an operationally useful but simplified concept describes macrophage polarization into classically (M1) and alternatively (M2) activated groups (5, 6). M1 macrophages produce inflammatory cytokines and reactive nitrogen or oxygen species that contribute to host defense, whereas M2 macrophages are poorly microbicidal and instead attenuate inflammation and participate in tissue remodeling (7). M1 macrophages are typically induced by granulocyte macrophage colony-stimulating factor (GM-CSF) together with IFN-γ and a microbial stimuli such as lipopolysaccharide (LPS), whereas macrophage colony-stimulating factor (M-CSF) and the Th2 cytokines, IL-4 and IL-13, have been shown to induce alternative M2 polarization providing a niche for survival of intracellular microbes (8, 9). Accordingly, mycobacterial virulence factors may interfere with M1 polarization and instead promote a phenotype switch toward alternatively activated M2 macrophages, which facilitate mycobacterial growth and intracellular persistence (10–12).

Alveolar macrophages are the resident macrophage population in the lung, and have been classified as alternatively activated M2-like cells due to their receptor expression and their role to regulate tissue homeostasis (13). Since TB is primarily a lung infection, the alveolar M2 macrophages may be the first cells to encounter and phagocytose mycobacteria (14). The local tissue environment is rich in MCSF that is produced by stromal cells in the steady state, favoring M2 activation of macrophages (15), while an infection could trigger GM-CSF as well as the other Th1 cytokines required for the induction of classically activated M1 macrophages (16). Along the spectrum of macrophage polarization, the immunomodulatory hormone vitamin D3 (vitD) may have significant effects on macrophage polarization and function. It has been shown that vitamin D is a potent inducer of the antimicrobial peptide LL-37 (17, 18) as well as autophagy (18, 19) in M-CSF polarized macrophages that correlates to enhanced intracellular inhibition of Mtb growth. Vitamin D is produced in the skin or ingested in the diet and converted in tissue by cells including macrophages to the active form 1,25(OH)_2_D_3_ (20). Active vitamin D signals via the intracellular vitD receptor (VDR) that act as a transcription factor complex to induce different host cell genes such as LL-37, and genes associated with autophagy. Mtb-induced toll-like receptor (TLR)-activation of human macrophages has been shown to trigger an up-regulation of the VDR as well as the vitamin D converting enzyme that results in a specific up-regulation of LL-37 and intracellular killing of Mtb (17). This way, vitamin D can induce VDR-dependent regulation of antimicrobial host responses in human macrophages.

In this study, we investigated the ability of 1,25(OH)_2_D_3_-polarized monocyte-derived cells to control intracellular Mtb infection compared with conventional M1 and M2 macrophage subsets, and the phenotypic as well as functional alterations associated with immune polarization *in vitro*. We demonstrate that 1,25(OH)_2_D_3_-polarized macrophage-like cells exhibit a superior effect on Mtb growth control compared with M1 and M2 macrophage subsets. Mtb infection of 1,25(OH)_2_D_3_-polarized cells resulted in a potent up-regulation of pro-inflammatory cytokines, LL-37 but also IL-10, while mRNA expression the IL-1β receptor antagonist, IL-1RA, and the immunosuppressive molecule indoleamine 2,3-dioxygenase (IDO), was down-regulated as compared with the other subsets. In conclusion, macrophage polarization in an environment rich in vitamin D may significantly enhance intracellular Mtb growth control, perhaps by inducing innate effector molecules and simultaneously regulating the anti-inflammatory response of the cell.

## Materials and Methods

### Monocyte Isolation and Differentiation

Peripheral blood mononuclear cells (PBMCs) from healthy donors (Karolinska Hospital Blood Bank, Stockholm, Sweden) were isolated from buffy coat blood by density sedimentation over Lymphoprep (GE Healthcare Life Sciences, Logan, UT). Cells were allowed to adhere in 6-well culture plates (Nunc, NY, Rochester, USA) for 2 h at 37°C in serum free media (RPMI 1640, supplemented with 2 mM L-glutamine and 5 mM HEPES (Hyclone, GE Healthcare Life Sciences). The non-adherent cells were removed by washing with phosphate buffered saline (PBS) and thereafter adherent monocytes were differentiated for 6 days in cell culture media containing 10% of fetal calf sera (FCS) (Sigma-Aldrich, St. Louis, MO). To establish an unbiased culture system that could assess the individual effects of 1,25(OH)_2_D_3_ compared to conventional M1 and M2 polarization methods, unstimulated control (M0) and 1,25(OH)_2_D_3_-polarized monocytes were differentiated in the absence of external growth factors using similar protocols as previously described (21, 22). As the maturation and activation status of macrophages differs depending on the *in vitro* stimulant (23), the cells obtained with the differentiation protocols used are referred to as monocyte-derived cells. Briefly, cells were left unstimulated or conditioned with 50 ng/mL human M-CSF (Life technologies, Carlsbad, CA) or 50 ng/mL human GM-CSF (ImmunoTools, Germany) for 3 days, after which the media was replaced with fresh media with or without M-CSF or GM-CSF (100 g/ml) for another 3 days of culture. To obtain 1,25(OH)_2_D_3_-polarized monocyte-derived cells or fully polarized M1 or M2 subsets, unstimulated monocyte cultures were treated with 10 nmol vitamin D [1,25(OH)_2_D_3_, Sigma-Aldrich], while GM-CSF differentiated monocytes were treated with 50 ng/mL interferon-gamma (IFN-γ) and 10 ng/mL lipopolysaccharide (LPS), and M-CSF differentiated monocytes were treated with 20 ng/mL IL-4 (ImmunoTools, Germany), 18–20 h prior to infection with Mtb. The macrophage polarization protocols (8) and nomenclature used in this study is summarized in Table 1. The morphology of monocyte-derived cell cultures was monitored with light microscopy and the viability of adherent cells after 7 days of culture was estimated to >95% using Trypan Blue (Sigma-Aldrich) staining.

**Table 1:** Polarization of monocyte-derived cells.

**Stimulation**	**Nomenclature^**a**^**
Unstimulated	M0 (Control)
Active vitamin D_3_	1,25(OH)_2_D_3_-polarized
GMCSF	M1-like
GMCSF + IFNγ + LPS	M1 polarized
MCSF	M2-like
MCSF + IL-4	M2 polarized

a *Nomenclature used in this study to describe differentially polarized monocyte-derived cell subsets*.

We used a fixed dose of 1,25(OH)_2_D_3_ (10 nmol) that was in a similar range as the doses previously used by us (18, 24) and other groups (17, 19) to study the *in vitro* effects of 1,25(OH)_2_D_3_ on human immune cells. The circulating 25(OH)D_3_ proform (10–100 nmol/L in serum) is converted to equivalent concentrations of the active 1,25(OH)_2_D_3_ form inside cells upon uptake of 25(OH)D_3_ (20), which is the basis for using the active form in the nanomolar range in cellular experiments, corresponding to a physiological concentration of active vitamin D.

### Mtb Cultures and Infection of Monocyte-Derived Cells

For infection of monocyte-derived cells, the standard laboratory Mtb strain, H37Rv, carrying the green fluorescent protein (GFP)-encoding pFPV2 plasmid (Mtb-GFP) was used (ATCC, Rockville, MD). A multidrug-resistant (MDR)-TB clinical isolate was obtained from the Mtb strain collection from the Public Health Agency of Sweden. The bacteria were stored at −80°C in Middlebrook 7H9 media supplemented with 10% glycerol (Karolinska University Hospital Solna, Sweden). Bacterial aliquots were thawed and cultured in Middlebrook 7H9 media, supplemented with 0.2% (v/v) glycerol, 10% OADC (Oleic Albumin Dextrose Catalase) enrichment and 0.05% (v/v) Tween-80 (Karolinska University Hospital Solna, Sweden) for 2–3 days. Bacteria were then sub-cultured in 50 ml tubes (dilution of bacterial stock 1:20 in new Middlebrook 7H9 media) and incubated at 37°C on a shaker for 7–10 days. Bacterial suspensions were pelleted and washed with phosphate buffer saline pH 7.4 (PBS) containing 0.05% (v/v) Tween-80 and then resuspended in 5 ml of RPMI complete media before transfer into a sterile 15 ml falcon tube. After 10 min pulse-sonication of inoculi, the optical density was measured at 600 nm. The final bacterial concentration was adjusted to ~2.5 × 10^6^ CFU/ml by adding RPMI complete media without antibiotics.

Primary monocytes were plated in 6-well plates at a concentration of 10^6^ cells per well and allowed to differentiate for 7 days before infection with Mtb for 4 h in 37°C at a multiplicity of infection (MOI) of 5. After infection, the cells were washed vigorously three times with PBS-0.05%Tween to remove extracellular bacteria and thereafter resuspended in cell culture media without antibiotics for further experiments and analyses.

### Flow Cytometry

Adherent Mtb-infected monocyte-derived cells were detached at 4, 24, or 72 h post-infection using 1.5 mM EDTA (Thermo Fisher Scientific Baltics UAB, Vilnius, Lithuania) for 10–15 min at room temperature. Detached cells (viability <90–95%) were washed with FACS buffer [PBS containing 0.5% (v/v) FCS and 0.5 mM EDTA] and 0.5–1 × 10^6^ cells were stained for 30 min at 4°C with fluorochrome-conjugated anti-human antibodies: CD68 (PE-Cy7), TLR2 (FITC), CD200R (PE), CD163 (BV605), CD206 (APC-Cy7), CD64 (PE-Dazzle 594), HLADR (PE-Cy5), CCR7 (BV711), CD80 (BV650), and CD86 (BV421) (Biolegend, San Diego, CA). Cells were washed twice with FACS buffer and fixed with 4% formaldehyde (Sigma-Aldrich) at room temperature for 10 min. Fifty thousand cells were acquired from each well by using BD LSR Fortessa (BD Biosciences, San Jose, CA) and analyzed with FlowJo v.9. H37Rv-GFP expression in CD68-positive macrophages was visualized in the FITC channel.

### Colony Forming Units (CFU)

On day 3 post-infection, monocyte-derived cell cultures were lysed with 0.036% SDS-lysis buffer (Sodium dodecyl sulfate from Sigma dissolved in water) for 5 min to release intracellular bacteria. Cell lysis was confirmed by fast-contrast microscopy. Standard CFU counts were determined by diluting samples 1/10 to 1/10,000 by mixing 100 μl of bacterial suspension in 900 μl PBS containing 0.05% (v/v) Tween-80 and finally plating a volume of 100 μl on Middlebrook 7H11 agar plates with 10% OADC (Karolinska University Hospital Solna, Sweden). Each dilution was performed in duplicates. The plates were incubated at 37°C for a minimum of 21 days before colonies were manually counted. As a negative control, supernatants from Mtb-infected cell cultures were collected before cell lysis and plated for CFU counts, which confirmed the absence of bacterial growth i.e., no extracellular Mtb bacilli remaining in Mtb-infected cell cultures.

### mRNA Extraction and Quantitative Real-Time PCR

Human lung tissue biopsies (TB lesion and distal sites), obtained from patients with non-cavitary TB (*n* = 5) as previously described (25, 26), were freeze-fixed in OCT (Tissue-TEK, Sakura) before cutting two 50 μm cryosections that were homogenized in 1 ml of TRI-reagent and processed for mRNA extraction. Similarly, RNA was extracted from uninfected or Mtb-infected monocyte-derived cells 24 h post-infection using Ribopure RNA extraction kit as described by the manufacturer (Ambion, Thermo Fisher Scientific, Waltham, MA). cDNA was synthesized using Super Script Vilo™ cDNA Synthesis Kit (Applied Biosystems, Foster City, CA). Transcripts of Arg1, Arg2, CAMP, NOS2A, CCL2, CCL22, TNFα, TGFβ, IL-10, TLR2, IL-6, IL-1β, IL-1RA, and IDO (Indoleamine 2,3-dioxygenase) (Applied Biosystems) were measured in duplicates relative to the reference gene, housekeeping 18S rRNA (18S rRNA-housekeeping gene kit, Applied Biosystems), using quantitative real-time PCT (RT-PCR) (QuantStudio 5 Real-Time PCR System, Thermo Fisher Scientific, MA, USA). The results were analyzed by using the relative standard method. Briefly, the relative expression of the target genes was calculated by relating the Ct-value for Mtb-infected to uninfected M0 monocyte-derived cells. Data are presented as fold change of mRNA.

### Statistical Analyses

Data are presented as the mean and standard deviation (SD) or median and interquartile range (IQR) from two or more individual experiments including monocyte-derived cells from a minimum of six healthy donors. The analyses used to calculate indicated *P*-values include one-way Anova, Kruskal–Wallis and Dunn's post-test as well as the Mann–Whitney or Wilcoxon-singed rank test. A *P* < 0.05^*^ was considered statistically significant. The statistical analyses were done in GraphPad Prism-6.

## Results

### Phenotype and Morphology of *in vitro* Polarized Monocyte-Derived Cells

**Figure 1:**
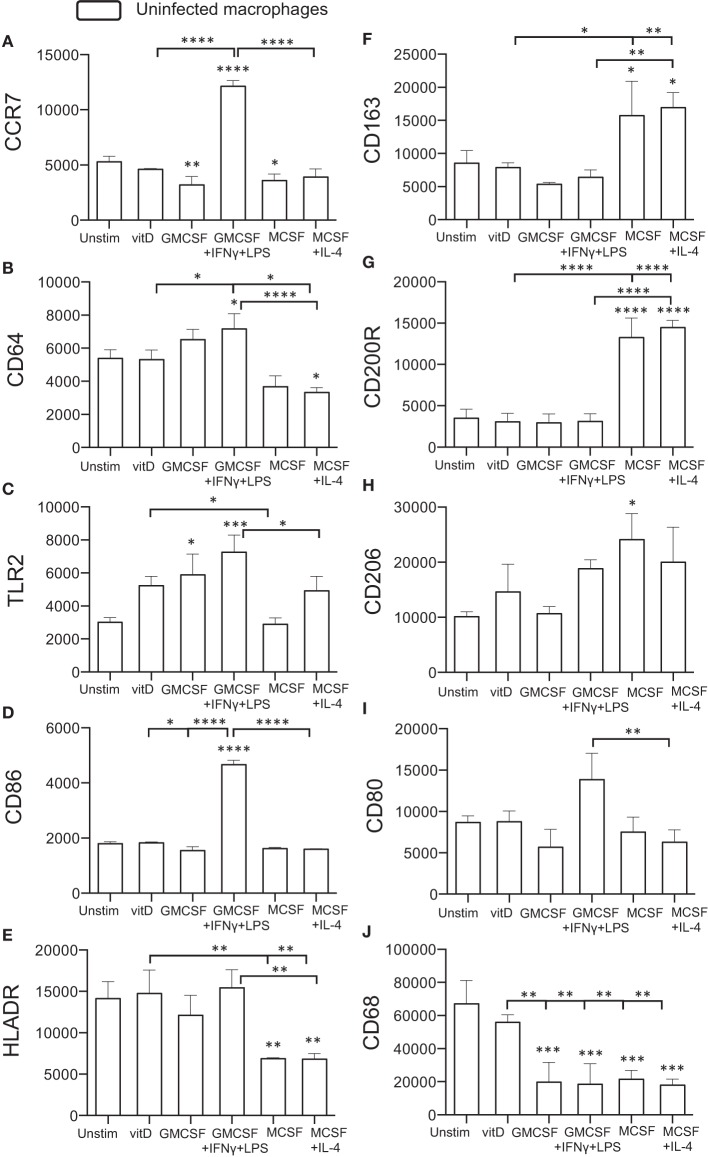
Surface expression of M1 and M2 markers on uninfected *in vitro* polarized macrophages was determined using flow cytometry. The left panel shows typical M1 markers **(A)** CCR7, **(B)** CD64, **(C)** TLR2, **(D)** CD86, and **(E)** HLADR, while the right panel shows typical M2 markers **(F)** CD163, **(G)** CD200R, **(H)** CD206, **(I)** CD80, or macrophage phenotype **(J)** CD68. Results were obtained from *n* = 6 donors. Data (mean fluorescence intensity, MFI) is presented as mean ± SD and was analyzed using one-way ANOVA, *p* < 0.05^*^, *p* < 0.01^**^, *p* < 0.001^***^, *p* < 0.0001^****^. For clarity, selected statistical analyses are shown including comparisons of the indicated group to the M0 control (no bars), or other relevant between group comparisons (half tick down lines).

Initially, surface expression of selected M1 (CCR7, CD64, TLR2) or M2 (CD163, CD200R, CD206) markers as well as antigen-presenting (HLADR) and co-stimulatory (CD86, CD80) molecules was investigated on uninfected monocyte-derived cells after immune polarization *in vitro*. CCR7, CD64, TLR2, and CD86 were all significantly (*P* < 0.05–0.0001) up-regulated on M1-polarized cells, and the expression of these markers were also relatively higher on M1-polarized compared with M1-like cells (Figures 1A–E). M2-polarized cell subsets had a down-regulated expression of CCR7, CD64, and HLA-DR (*P* < 0.05–0.001) compared with unstimulated M0 cells (Figures 1A,B,E) and also a significantly (*P* < 0.01) lower expression of CD80 compared to M1-polarized cells (Figure 1I). Instead, M2-like and M2-polarized cells showed a significant up-regulation of the M2 markers CD163 (*P* < 0.05), CD200R (*P* < 0.0001), and CD206 (*P* < 0.05) (Figures 1F–H). 1,25(OH)_2_D_3_-polarized cells up-regulated TLR2, CD64 and HLADR (*P* < 0.05–0.01) compared with the M2 subsets (Figures 1B,C,E) and simultaneously showed a lower expression of CD163 and CD200R (*P* < 0.05–0.0001) (Figures 1F,G). In comparison with the M1 subsets, 1,25(OH)_2_D_3_-polarization resulted in a lower expression of CCR7, CD64, CD86 (*P* < 0.05–0.0001) (Figures 1A,B,D). Similar to M0 cells, 1,25(OH)_2_D_3_-polarized cells had a relatively higher expression of CD68 compared with M1 as well as M2 polarized cell subsets (Figure 1J). Thus, polarization of uninfected monocyte-derived cells toward M1 or M2 resulted in up- or down-regulation of typical M1/M2 surface markers, while 1,25(OH)_2_D_3_-polarization did not promote either M1 or M2 differentiation.

**Figure 2:**
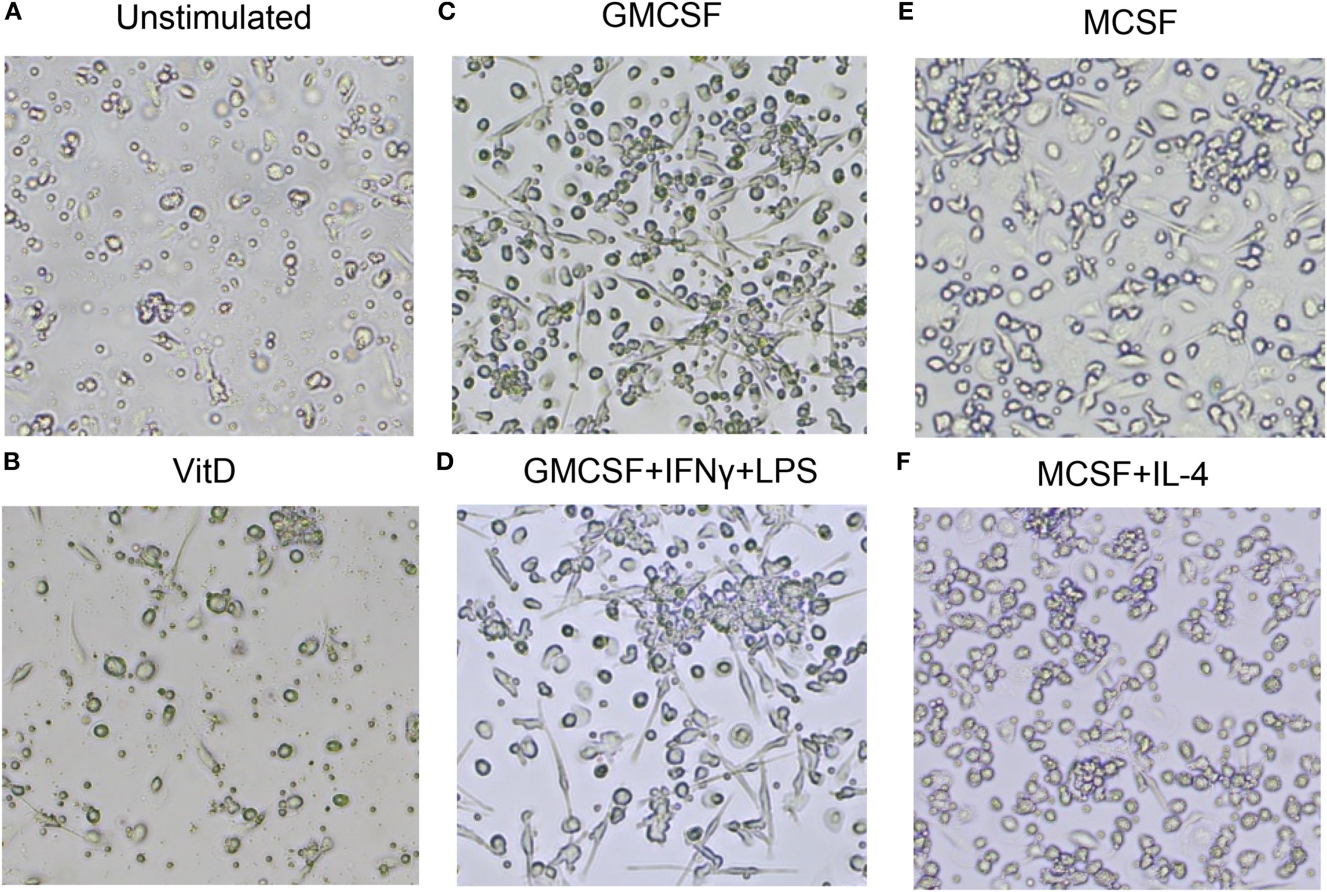
Morphology of uninfected macrophage subsets after *in vitro* polarization. **(A)** Unstimulated M0 cells, **(B)** 1,25(OH)_2_D_3_-polarized cells, **(C)** GMCSF-polarized M1-like macrophages, **(D)** GMCSF + IFNγ + LPS M1-polarized macrophages, **(E)** MCSF-polarized M2-like macrophages, **(F)** MCSF+IL-4 M2-polarized macrophages. Magnification 20×.

Next, light microscopy was used to study the morphology of immune polarized monocyte cultures (Figure 2). Unstimulated 1,25(OH)_2_D_3_-polarized cells showed a more circular morphology compared with M1 and M2 cells (Figures 2A–F). As expected, M1-like and M1-polarized cells had a more elongated and stretched or dendritic-like morphology compared with the other subsets (Figures 2C,D). M2 subsets were confluent but with a rounded shape, and M2-polarized cells were more circular compared with M2-like cells (Figures 2E,F). Overall, these results confirmed that M1 and M2 subsets had a phenotype and morphology consistent with classically and alternatively activated macrophages, respectively. Instead, 1,25(OH)_2_D_3_-polarized cells maintained the morphology of less mature macrophages, but a high surface expression of CD68, CD64, and HLADR were consistent with differentiation of monocyte-derived macrophage-like cells.

### Uptake and Intracellular Growth of Mtb in *in vitro* Polarized Monocyte-Derived Cells

**Figure 3:**
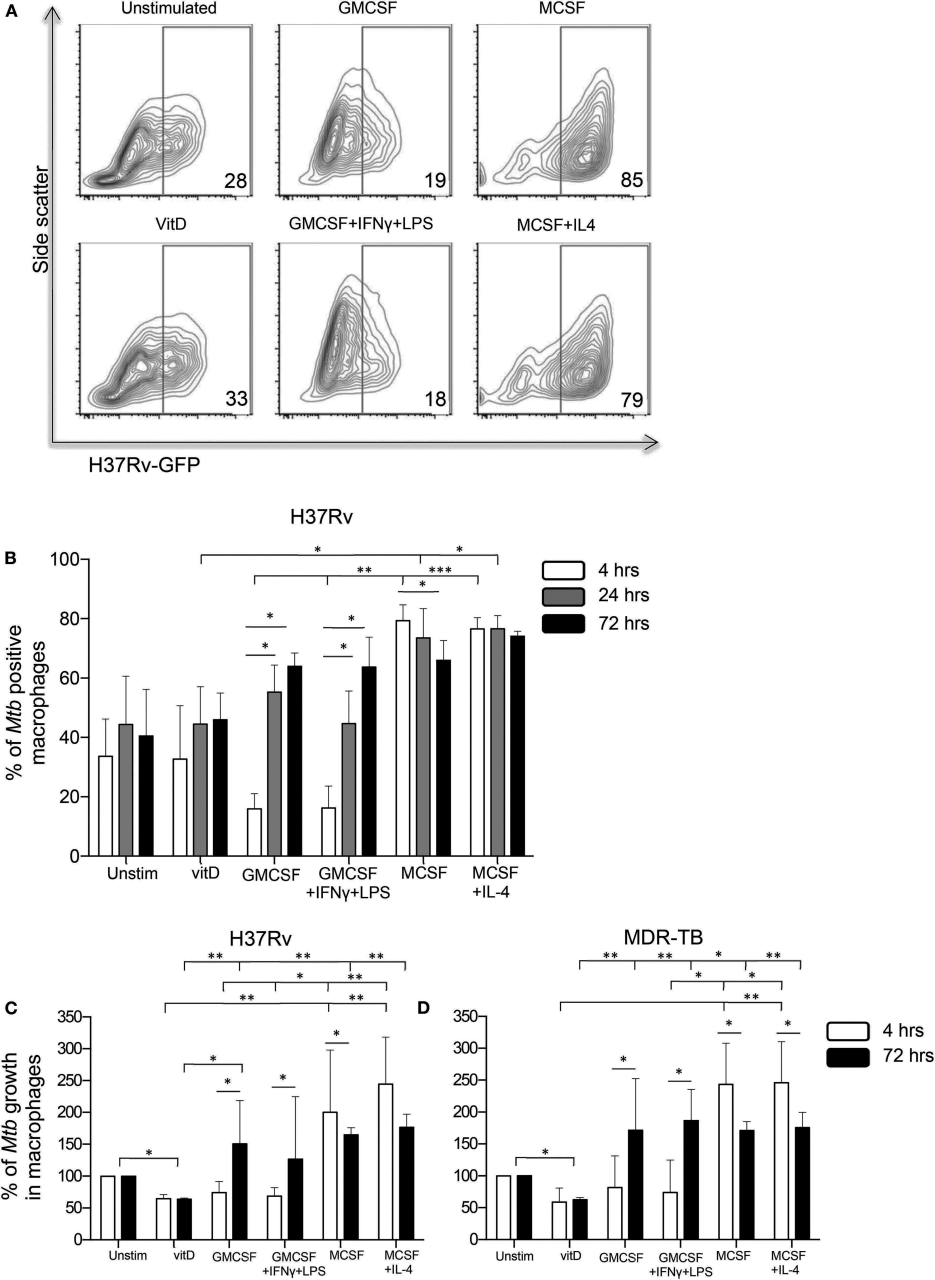
Uptake, infectivity and intracellular growth of Mtb in *in vitro* polarized macrophages after Mtb infection. Flow cytometry was used to assess intracellular GFP-labeled H37Rv in *in vitro* polarized macrophage subsets at 4, 24, and 72 h post-infection. **(A)** Representative contour plots show the expression of H37Rv-GFP in the FITC-channel at 4 h post-infection. The percentage GFP-positive cells are indicated in the corner of each plot. **(B)** Mtb uptake in *in vitro* polarized macrophages at 4 h (white bars) compared to productive infection at 24 h (gray bars) and at 72 h (black bars) post-infection with H37Rv-GFP. Bacterial uptake and infection are presented as % of Mtb positive macrophages (% H37Rv-GFP positive cells). Intracellular growth of **(C)** H37Rv and **(D)** an MDR-TB clinical isolate in *in vitro* polarized macrophage subsets determined at 4 h (white bars) and 72 h (black bars) post-infection using CFU counts. Intracellular growth is presented as % of Mtb growth in macrophages (unstimulated M0 control = 100% growth). Results were obtained from *n* = 6 donors. Data is presented as median ± IQR and was analyzed using the Kruskal-Wallis and Dunn's post-test (comparing different polarization conditions), Friedman's test (comparing 4, 24, and 72 h time-points) and Wilcoxon signed-rank test (comparing 4 h to 72 h), *p* < 0.05^*^, *p* < 0.01 ^**^, *p* < 0.001^***^.

To explore if vitamin D could affect Mtb uptake and subsequently a productive infection of *in vitro* polarized monocyte-derived cells, expression of GFP-labeled Mtb (H37Rv) was assessed after 4 h (uptake), 24 and 72 h (productive infection) post-infection of the different subsets. At 4 h, we observed a relatively lower percentage of M1-like and M1-polarized cells expressing H37Rv-GFP compared with the other subsets (Figures 3A,B), and this difference was significant comparing the M1 subsets with the M2 subsets (*P* < 0.01–0.001) (Figure 3B). Mtb uptake was evident in 18–19% of M1 cells, while ~30% of unstimulated M0 as well as 1,25(OH)_2_D_3_-polarized cells, and about 82% of M2 cells, were H37Rv-GFP-postive at 4 h (Figures 3A,B). However, at 24 and 72 h post-Mtb infection, the level of GFP-expressing cells rapidly and gradually increased in the M1 subsets (*P* < 0.05), while Mtb infectivity declined in M2-like cells (*P* < 0.05) and remained unchanged in the other monocyte-derived cell subsets (Figure 3B). At 24 h, the level of productive Mtb infection was significantly lower in 1,25(OH)_2_D_3_-polarized cells compared with the M2-like and M2-polarized cells (*P* < 0.05) (Figure 3B).

Next, intracellular growth of Mtb was assessed at 4 and 72 h post-infection with H37Rv (Figure 3C) or a clinical MDR-TB isolate (Figure 3D) using CFU counts. At 4 h post-infection, we observed substantially elevated CFU counts in the M2 subsets compared with the 1,25(OH)_2_D_3_ and M1 groups (*P* < 0.05–0.01) (Figures 3C,D), indicating a reduced colony forming capacity of Mtb bacilli that has been taken up by 1,25(OH)_2_D_3_-polarized or M1 cells. This could result from enhanced bacterial killing or reduced metabolic activity (i.e., latency) of the bacteria. Despite a lower Mtb uptake in M1 cells compared with 1,25(OH)_2_D_3_-polarized cells (Figure 3B), the colony forming capacity was similar in these monocyte-derived cell subsets at 4 h (Figures 3C,D). Consistent with the FACS-data, significantly (*P* < 0.05–0.01) enhanced intracellular bacterial growth was detected in the M1- as well as M2-polarized subsets compared with 1,25(OH)_2_D_3_-polarized cells, 72 h post-infection (Figures 3C,D). Notably, Mtb growth in the 1,25(OH)_2_D_3_-polarized subset was around 40% lower compared with unstimulated M0 cells (*P* < 0.05), while Mtb growth was around 100–200% higher in the M1 and M2 subsets at 72 h (Figures 3C,D). Accordingly, intracellular Mtb growth was maintained at relatively lower levels in 1,25(OH)_2_D_3_-polarized compared with unstimulated M0 cells, while there was substantial growth in both M1 and M2 cells (Figures 3C,D).

### Alterations of M1/M2-Specific Markers Expressed on *in vitro* Polarized Monocyte-Derived Cell Subsets After Mtb Infection

**Figure 4:**
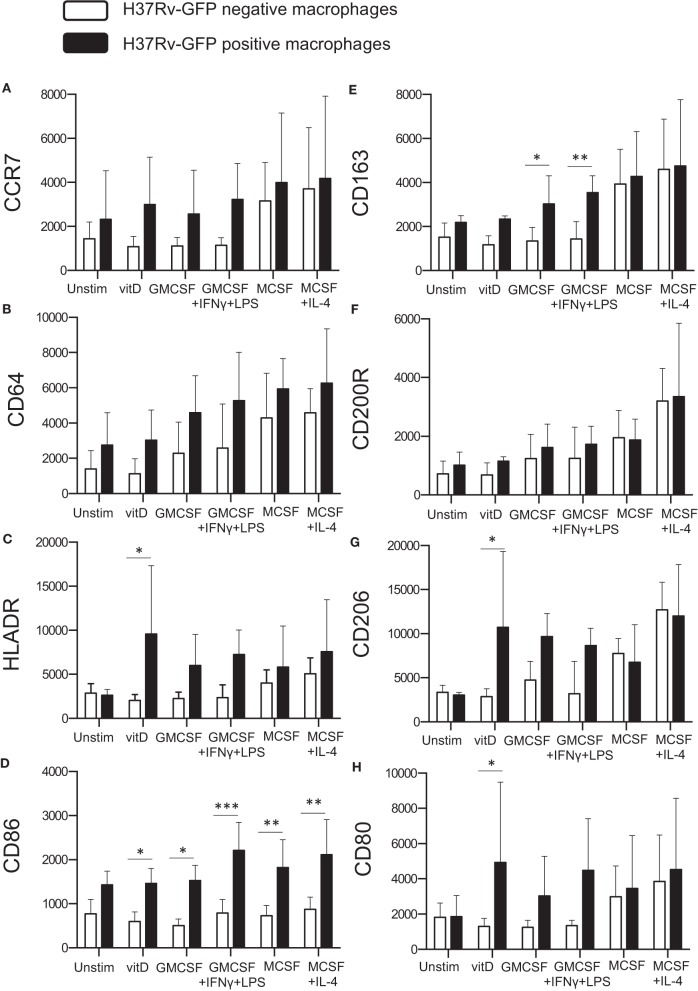
Surface expression of M1 and M2 markers on Mtb-infected *in vitro* polarized macrophages was determined using flow cytometry. H37Rv-GFP positive (black bars) and H37Rv-GFP negative (white bars) cells were assessed in the sample. The left panel shows M1 markers **(A)** CCR7, **(B)** CD64, **(C)** HLA-DR, and **(D)** CD86, while the right panel shows M2 markers **(E)** CD163, **(F)** CD200R, **(G)** CD206, and **(H)** CD80. Results were obtained from *n* = 6 donors. Data (mean fluorescence intensity, MFI) is presented as mean ± SD and was analyzed using two-way ANOVA, *p* < 0.05^*^, *p* < 0.01^**^, *p* < 0.001^***^.

Next, we assessed surface expression of the tested panel of M1/M2-specific markers on *in vitro* polarized monocyte-derived cells 4 h after infection with H37Rv-GFP. Cells in the same sample were divided into GFP-positive (Mtb-infected) and GFP-negative (uninfected) expression. Interestingly, Mtb infection altered the expression of typical M1 phenotype markers (Figures 1A–D), which were all elevated on M2 compared with M1 cell subsets (Figures 4A–D). Instead, the M2 marker CD163 was significantly up-regulated on GFP-positive M1-like (*P* < 0.05) and M1-polarized (*P* < 0.01) cells, respectively (Figure 4E). All H37Rv-GFP-positive subsets but M2, exhibited a relative up-regulation of all markers except CD200R (Figure 4). Furthermore, CD86 was significantly (*P* < 0.05–0.001) up-regulated on all GFP-positive subsets but unstimulated M0 cells (Figure 4D). 1,25(OH)_2_D_3_-polarized cells also displayed a significant (*P* < 0.05) up-regulation of HLA-DR (Figure 4C), the mannose receptor CD206 (Figure 4G) and CD80 (Figure 4H). Overall, these results suggest that Mtb-infected monocyte-derived cell subsets experience phenotypical alterations suggestive of a mixed M1/M2 profile.

Assessment of kinetic changes of M1/M2-specific markers on M2-like macrophages, revealed a rapid up-regulation of surface markers at 4–12 h post-Mtb infection that declined already at 24–36 h after infection (Supplementary Figure 1). At 24 and 72 h post-Mtb infection, there were no significant differences in the expression of M1 or M2 markers between GFP-positive and GFP-negative cells or comparing the different subsets (data not shown).

### Transcriptional Profiling of *in vitro* Polarized Monocyte-Derived Cells After Mtb Infection

**Figure 5:**
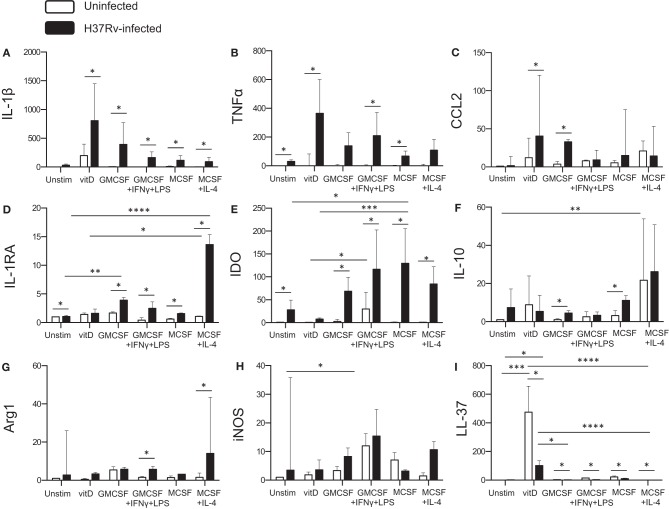
Quantitative mRNA expression in Mtb-infected *in vitro* polarized macrophages was determined at 24 h post-infection. Different effector molecules including pro- or anti-inflammatory cytokines, chemokines, inhibitory receptors, and antimicrobial effector molecules were assessed in uninfected compared to Mtb-infected macrophage subsets. **(A)** IL-1β, **(B)** TNF-α, **(C)** CCL2, **(D)** IL-1RA, **(E)** IDO, **(F)** IL-10, **(G)** Arg-1, **(H)** iNOS, and **(I)** LL-37. mRNA expression was determined using RT-PCR and presented as the fold change of each target gene in the different Mtb-infected subsets compared to unstimulated M0 macrophages. Results were obtained from *n* = 9 donors. Data is presented as median ± interquartile range and analyzed using the Kruskal-Wallis and Dunn's post-test (comparing different polarization conditions), or Wilcoxon signed-rank test (comparing H37Rv-infected to uninfected cells), *p* < 0.05^*^, *p* < 0.01 ^**^, *p* < 0.001^***^, *p* < 0.0001^****^.

To obtain additional information of the functional properties of *in vitro* polarized monocyte-derived cell subsets, quantitative mRNA analyses were performed on Mtb-infected cells 24 h post-infection and compared with uninfected cells. Mtb infection induced production of pro-inflammatory cytokines, IL-1β and TNF-α (Figures 5A,B) or IL-6 (data not shown), in the different subsets (*P* < 0.05), although both IL-1β and TNF-α was relatively higher in 1,25(OH)_2_D_3_-polarized cells (Figures 5A,B). Moreover, Mtb enhanced expression of the monocyte chemoattractant CCL2 in 1,25(OH)_2_D_3_-polarized cells, but also in the M1-like subset (*P* < 0.05) (Figure 5C). Instead, the IL-1β antagonist, IL-1RA, was significantly induced in Mtb-infected M2-polarized subset (*P* < 0.0001) compared with the unstimulated control (Figure 5D). All other Mtb-infected monocyte-derived cell subsets, apart from 1,25(OH)_2_D_3_-polarized cells, also had a significant up-regulation of IL-1RA (*P* < 0.05) (Figure 5D). Similarly, Mtb infection induced a high mRNA expression of the immunosuppressive enzyme IDO, in M0, M1 as well as the M2 subsets (*P* < 0.05) (Figure 5E). Contrary, IDO mRNA was very low in 1,25(OH)_2_D_3_-polarized cells (Figure 5E). IDO mRNA was also induced in uninfected M1-polarized cells, which is consistent with the finding that IFN-γ up-regulates IDO (27). IL-10 mRNA was induced in both 1,25(OH)_2_D_3_ and M2-polarized uninfected cells (*P* < 0.001) and increased in M1-like as well as M2-like cells (*P* < 0.05) after Mtb infection (Figure 5F). mRNA expression of the intracellular enzymes arginase, Arg-1 (Figure 5G) and Arg-2 (data not shown), was primarily observed in M1- as well as M2-polarized subsets post-Mtb infection (*P* < 0.05), whereas inducible nitric oxide (iNOS) was relatively higher in uninfected as well as Mtb-infected M1 cells (*P* < 0.05) (Figure 5H). Mtb also induced iNOS in M2-polarized cells and to a lesser extent in 1,25(OH)_2_D_3_-polarized cells (Figure 5H). As expected, mRNA induction of the antimicrobial peptide, LL-37, was superior in 1,25(OH)_2_D_3_-polarized monocyte-derived cells (*P* < 0.001). Mtb infection resulted in a potent down-regulation of LL-37 expression in all subsets (*P* < 0.05) (Figure 5I), but was still significantly higher in 1,25(OH)_2_D_3_-stimulated compared with M1-like (*P* < 0.05) and M2-polarized (*P* < 0.0001) subsets (Figure 5I). LL-37 was not induced in unstimulated M0 cells (Figure 5I). Likewise, the autophagy markers LC3B as well as Atg5 and beclin-1 (data not shown) were selectively up-regulated in 1,25(OH)_2_D_3_-polarized cells. mRNA expression profiles in monocyte-derived cells infected with H37Rv or the clinical MDR-TB isolate were very similar and revealed no significant differences between these mycobacterial strains (data not shown).

Overall, Mtb infection resulted in enhanced or unchanged transcription of the different molecules investigated, with the exception from LL-37 mRNA expression that was significantly suppressed by Mtb infection. Corresponding mRNA profiling of lung tissue samples obtained from patients with non-cavitary TB, confirmed that most of the investigated markers were up-regulated in the TB lesions (site of Mtb infection) compared with unaffected lung parenchyma, supporting the presence of a mixed M1/M2 macrophage polarization *in vivo* (Supplementary Figure 2).

## Discussion

Immune polarization of macrophages in the local tissue environment may have significant effects on the ability of the cells to control intracellular Mtb infection. We demonstrate that monocyte-derived cells polarized with the active form of vitamin D have a superior effect on Mtb growth inhibition in comparison to cell subsets polarized using conventional M1 and M2 stimuli. While uninfected M1 and M2 subsets expressed typical phenotype markers, Mtb infection altered this expression and generated a mixed M1/M2 activation profile. Mtb infection of M1 cells was initially low but rapidly progressed into a productive infection and enhanced bacterial growth, while Mtb growth remained low in 1,25(OH)_2_D_3_-polarized cells. Contrary, infectivity of and Mtb growth in the M2 subsets was high 4 h post-infection but subsequently decreased to levels comparable to M1 cells supporting the mixed M1/M2 model. Mtb growth inhibition in 1,25(OH)_2_D_3_-polarized cells was associated with an induction of the antimicrobial peptide LL-37 as well as pro-inflammatory cytokines but also IL-10 mRNA, while expression of IL-1RA and IDO was low in comparison to the other monocyte-derived cell subsets. Our data demonstrates that polarization of monocyte-derived cells in the presence of vitamin D provides the capacity to mount an antimicrobial response against Mtb that is superior to conventional M1 and M2 activation, and that simultaneously prevent expression of inhibitory molecules that could promote immunosuppression in the microenvironment of Mtb-infected tissues.

We have previously conducted human studies to investigate the relevance of vitamin D status in the progression of clinical TB (26, 28), the *in vitro* effects of vitamin D stimulation on monocyte-derived macrophages (MDMs) (18, 24) and the clinical benefit from *in vivo* supplementation of vitamin D together with similar immunomodulatory compounds to patients with pulmonary TB (29, 30). Our findings showed that MDMs obtained from TB patients produced increased amounts of IL-1β, TNF-α as well as IL-10 upon stimulation with 1,25(OH)_2_D_3_, which were associated to reduced growth of intracellular Mtb (24). Furthermore, 1,25(OH)_2_D_3_, in the presence or absence of phenylbutyrate (PBA), had the ability to counteract Mtb-induced down-regulation of LL-37 in MCSF-differentiated macrophages and could also induce autophagy via a LL-37-dependent pathway that promoted intracellular growth control of Mtb (18). Vitamin D deficiency is common in patients with TB, although the cause and effect of low vitamin D status and the development of active TB is not known (20). Nonetheless, several studies provide evidence that LL-37 mRNA and protein expression in different macrophage subsets is dependent on vitamin D status (17, 31, 32). We have shown that a low expression of LL-37 in granulomatous TB lesions from patients with active TB correlates with low plasma levels of vitamin D [25(OH)D_3_] (26, 28). In clinical trials (29, 30), we have also observed positive clinical effects using 2–4 months adjunct treatment with vitD + PBA that resulted in improved vitamin D status and enhanced sputum-culture conversion in patients with pulmonary TB. Patient recovery was associated with up-regulated levels of LL-37 in MDMs and PBMCs (29) as well as induction of autophagy and reduced endoplasmic reticulum (ER)-stress in *ex vivo* Mtb-infected macrophages from vitamin D-supplemented TB patients (33). Accordingly, serum from vitamin D deficient individuals failed to support induction of LL-37 in human monocytes, while *in vitro* supplementation with 25(OH)D_3_ promoted LL-37 expression significantly (17). Similarly, vitamin D deficient serum prevented IFN-γ induced activation of LL-37 or β-defensin-4 mRNA in monocytes or macrophages that was restored by addition of 25(OH)D_3_ to the cell cultures (32). As a consequence, IFN-γ induced autophagy and phagolysosome fusion in monocytes as well as Mtb killing in MDMs were dependent on adequate levels of vitamin D (32). Another report revealed that Mtb and its secreted components can prevent the IFNγ signaling pathway and Mtb killing in infected macrophages by inhibition of IFNγ transcriptional responses (34). Notably, one of IFN-γ transcriptional targets is the vitamin D converting enzyme, 1α-hydroxylase (32). Altogether, these data suggest that vitamin D status including sufficient vitamin D concentrations locally in the tissue microenvironment may be of fundamental importance to trigger anti-TB defense pathways in human macrophages and can also contribute to the regulation of IFN-γ signaling.

Similar to our results, it has previously been demonstrated that M1-like macrophages were less susceptible to uptake and infection with avirulent *M. bovis* BCG compared with M2-like macrophages (35). However, BCG-infected M1 cells maintained enhanced intracellular growth control compared with M2 cells up to 6 days post-infection (35), suggesting that the elevated infectivity and Mtb growth observed in M1 cells rapidly after infection is a trait of virulent mycobacteria. A recent study determined that M2 macrophages displayed slightly higher phagocytic activity than M1 cells, and phagocytosis was not changed by treatment with 1,25(OH)_2_D_3_ (36). Furthermore, MDMs treated with low-dose 1,25(OH)_2_D_3_ for 6 days were less infected with dengue virus compared with untreated MDMs, which was proposed to be due to reduced viral uptake via CD206 that was down-regulated on 1,25(OH)_2_D_3_-tretated MDMs (37). In contrast, we found that CD206 was up-regulated on Mtb-infected monocyte-derived cells that were polarized with a higher dose of 1,25(OH)_2_D_3_ the last 20 h of 6 days differentiation. An enhanced expression of CD206 together with HLADR, CD86, and CD80 on 1,25(OH)_2_D_3_-polarized cells could suggest that antigen-presentation and co-stimulation is improved by vitamin D. Interestingly, also Chlamydia infection of polarized MDMs (8) or murine bone-marrow derived macrophages (38) was considerably lower in M1 compared with M2 cells. However, despite microbicidal activity, M1 macrophages failed to eliminate intracellular infection, but suppressed Chlamydia growth correlated with the induction of a bacterial gene expression profile characteristic of persistence (38). This is consistent with our results that Mtb-infected M1 cells were initially very potent to control infection but fail to completely eradicate intracellular mycobacteria that instead can persist in M1 as well as M2 macrophages.

The strength of our results is that we have used virulent Mtb and compared infectivity and intracellular Mtb survival over time in differentially polarized human monocyte-derived cell subsets. We also observed the plasticity of polarized cells, as Mtb infection generated a mixed M1/M2 phenotype. While *in vitro* studies suggest that human monocytes can polarize into M1 macrophages and subsequently switch to the M2 phenotype upon exposure to sequential changes in the microenvironmental conditions (39), different tissues *in vivo* i.e., the lung, may contain varying mixtures of M1- and M2-type macrophages (40). As such, macrophages in skin (41) or brain (41) tissue from patients with cancer or multiples sclerosis, respectively, present heterogeneous activation states more consistent with poly-activated macrophages. Mixed M1 and M2 activation phenotypes was recently shown to be induced by melanoma exosomes, suggesting that subcellular fractions and soluble factors can promote mixed M1/M2 activation (42). This could explain the altered phenotype we observed in Mtb-GFP-positive as well as Mtb-GFP-negative polarized monocyte-derived cells. Altogether, instead of shifting M1 toward M2 activation in Mtb infection, our findings support the mixed M1/M2 model that may facilitate intracellular persistence of Mtb.

The ability of macrophages to acquire different functional phenotypes enables them to execute innate effector functions but also to control the induction of specific T cell responses. After exposure to *M. bovis* BCG and subsequent antigen presentation to CD4^+^ T cell clones, M1 macrophages were more effective than M2 cells to stimulate proliferation and IFN-γ production (35). Expression of the immunosuppressive enzyme IDO in Mtb-infected cells effectively diminish activation of Th1 cells by degradation of the essential amino acid tryptophan, and may instead produce metabolites that induce FoxP3^+^ regulatory T cells (Treg) (43, 44). Our observations suggest that IDO was strongly up-regulated by Mtb infection in all conventionally polarized cell subsets, but to a much lesser extent in 1,25(OH)_2_D_3_-polarized monocyte-derived cells. Increased levels of IDO have been observed in TB patients (45, 46) and IDO can be expressed in Mtb-infected macrophages *in vitro* (46). Interestingly, IDO activity has been implicated in the differentiation of monocytes into M2 macrophages (47). Accordingly, ectopic IDO increased the expression of M2 markers but decreased M1 markers, while knock-down of IDO had the opposite effects (27). This suggests that IDO-expressing macrophages may skew differentiation toward the M2 phenotype. Accordingly, studies in non-human primates have shown that *in vivo* inhibition of IDO expression promoted local recruitment of effector T cells to the site of Mtb infection in lung granulomas and reduced bacterial burden significantly (48).

A limitation of this study is the use of an *in vitro* system based on Mtb infection of monocyte-derived cells that may only partially reproduce the complexity of macrophage activation found *in vivo*. However, it is likely that monocytes are recruited from the blood into the lung upon Mtb infection and develop into inflammatory MDMs including the M1 subsets that support tissue-resident macrophages in the combat against the bacteria (40). CCL2 is one of the main chemokines involved in inflammation-dependent recruitment of monocytes to sites of infection (49). Vitamin D-mediated induction of IL-10 could, similarly to IL-10 produced by M2 macrophages, be involved in resolution of inflammation and prevent overt immunopathology in Mtb-infected tissue, as has been described in vitamin D-supplemented TB patients (50).

This study provides new information about the potential local effects of vitamin D in the microenvironment of Mtb-infected tissues e.g., in the lung. Vitamin D is multifunctional molecule that has a broad range of effects on the immune response, and thus more research is required to obtain a comprehensive understanding of the how vitamin D status and vitamin D supplementation could contribute to TB control in patients. While we and others have tested the therapeutic capacity of vitamin D (29, 30), large clinical trials is currently ongoing in an attempt to study the prophylactic effects of vitamin D in preventing development of active TB infection [reviewed in (20)]. Overall, additional *in vitro* and *in vivo* studies will shed light on the role of vitamin D in macrophage polarization and if improved vitamin D status could enhance antimicrobial effector responses and simultaneously ameliorate inflammation without inducing immunosuppression in human TB.

## Data Availability Statement

All datasets generated for this study are included in the article/Supplementary Material.

## Ethics Statement

The studies on immune cells in blood obtained from healthy individuals was reviewed and approved by the ethical review board (EPN) in Stockholm, Sweden (2010/603-31/4). Written informed consent for participation was not required for this study in accordance with the national legislation and the institutional requirements.

## Author Contributions

SB, ML, and JR designed the research. JR and VP performed the experiments. JR, VP, ML, MS, and SB contributed to data analyses and interpretation. SB and JR wrote the manuscript. JR, VP, ML, MS, and SB critically reviewed and approved the final version of the manuscript.

### Conflict of Interest

The authors declare that the research was conducted in the absence of any commercial or financial relationships that could be construed as a potential conflict of interest.
