# Supplementary material for: Polarization of Human Monocyte-Derived Cells With Vitamin D Promotes Control of Mycobacterium tuberculosis Infection
Source: Front Immunol. 2020 Jan 22;10:3157. doi: 10.3389/fimmu.2019.03157 (PMC6987394; doi:10.3389/fimmu.2019.03157)
Supplement: Supplementary file 1 [file Data_Sheet_1.docx]

Supplementary Material

# Supplementary Figures

**A.**

**B.**

**C.**

CCR7

HLADR

CD86

CD163

CD200R

CD206

**D.**

**E.**

**F.**

CD163

CD200R

CD80

Uninfected

H37Ra

**Supplementary Figure 1.** Surface expression of different M1 and M2 markers on H37Ra-infected compared to uninfected M2-like polarized macrophages (MCSF) was determined using flow cytometry. (A) CCR7, (B) HLA-DR, (C) CD86, (D) CD163, (E) CD200R, (F) CD80. Data (mean fluorescence intensity, MFI) is presented as median ± IQR at 4, 12, 24 and 36 hours (hrs) post-infection with Mtb.

**Supplementary Figure 2.** Quantitative mRNA expression of different immune molecules in lung tissue biopsies obtained from patients with non-cavitary pulmonary TB. mRNA expression was determined using RT-PCR and presented as the fold change of each target gene in pathological TB lesions compared to unaffected lung parenchyma from the same patient (n=5). Data (fold change) is presented as median ± IQR.
